# A risk factor prediction model for moderate-to-severe postoperative pain in patients undergoing laparoscopic sleeve gastrectomy

**DOI:** 10.1097/MD.0000000000041398

**Published:** 2025-02-07

**Authors:** Yaning Yang, Chengzhen Zhang, Wenying Chi, Bin Zheng, Xiaoqian Yu, Kaiyun Zhang, Guo Junzuo, Fanjun Meng

**Affiliations:** a School of Anesthesiology, Shandong Second Medical University, Weifang, China; b Department of Anesthesiology, Central Hospital Affiliated to Shandong First Medical University, Shandong, PR China; c Department of Anesthesiology, Shandong First Medical University, Jinan, Shandong, PR China; d Hospital of Shandong Technology and Business University, Yantai, China.

**Keywords:** body mass index, laparoscopic sleeve gastrectomy, moderate-to-severe pain, modified frailty index, risk factor prediction model

## Abstract

The primary goal of this study was to identify the risk factors contributing to moderate-to-severe postoperative pain in patients undergoing laparoscopic sleeve gastrectomy (LSG) and to create a predictive model for these risk factors. A retrospective analysis was performed on a cohort of 375 patients who underwent LSG at Jinan Central Hospital from January 2017 to June 2023. Data for this study was extracted using medical databases. Patients were classified into 2 groups based on their postoperative pain levels: those experiencing moderate-to-severe pain and those not experiencing moderate-to-severe pain. Univariate and multivariate logistic regression analyses were employed to determine which variables were significantly associated with moderate-to-severe pain. Receiver operating characteristic curves were utilized to assess the diagnostic efficacy of different indicators. Additionally, calibration curves and clinical decision curves were applied for model validation. Multifactorial logistic regression analysis identified age, body mass index (BMI), and the modified frailty index (mFI) as independent risk factors for moderate-to-severe postoperative pain in LSG patients. Based on the regression analysis, a predictive model was constructed. The receiver operating characteristic curve for this model demonstrated an area under the curve of 0.96 (95% CI: 0.94–0.97), indicating excellent discriminatory ability between patients likely and unlikely to experience moderate-to-severe pain post-surgery. A scoring system was developed from the predictive model, assigning points to each risk factor. BMI was the most significant predictor (100 points), followed by mFI (30 points) and age (15 points). Calibration analysis showed that the predicted values closely matched the actual values, with a mean error of 0.008, indicating high accuracy of the model. Clinical decision analysis demonstrated a positive net benefit when the threshold probability ranged from 0.001 to 0.999, suggesting broad applicability of the model in clinical decision-making. Age, BMI, and mFI are significant predictors of moderate-to-severe postoperative pain in patients undergoing LSG.

## 1. Introduction

Obesity is a significant global health concern, with a high prevalence and a strong association with numerous chronic diseases, including cardiovascular disease, diabetes mellitus, chronic kidney disease, nonalcoholic fatty liver disease, metabolic syndrome, and various forms of cancer.^[1]^ In recent years, there has been a marked increase in the prevalence of obesity in many countries. China has now become the country with the highest prevalence of overweight and obese individuals in the world, with an adult overweight rate of 34.3% and an obesity rate of 16.4%.^[2,3]^ Concurrently, the prevalence of obesity is on the rise among the elderly population.^[2]^ Morbid obesity is frequently accompanied by a range of metabolic abnormalities, including type 2 diabetes, lipid metabolism disorders, and hypertension. In order to address the challenges associated with obesity and its related diseases, bariatric metabolic surgery is recommended by international guidelines as a standard treatment option for patients with obesity combined with metabolic diseases or weight-related complications such as hypertension and sleep apnea.^[1]^ Among these, laparoscopic sleeve gastrectomy (LSG) is one of the most commonly employed surgical interventions for the treatment of morbid obesity, due to its technical feasibility, substantial weight loss, improved comorbidities, and safety profile.^[4]^ Furthermore, in the context of perioperative anesthetic management, our findings indicated that despite the utilization of standardized intraoperative anesthetic management and postoperative analgesic regimens, the prevalence of moderate-to-severe postoperative pain was higher in patients undergoing LSG than in patients undergoing other types of laparoscopic surgery. Additionally, there were notable variations in the degree of postoperative pain experienced by patients with LSG, contingent on their body weights and preoperative statuses. Despite the many advantages of LSG, various postoperative complications have been observed, such as anastomotic leakage, bleeding, postoperative pain, gastroesophageal reflux disease, and postoperative nausea and vomiting (PONV).^[4–6]^

Pain sensitivity is elevated in obese patients compared to normal, and many patients will experience moderate-to-severe pain after laparoscopy.^[7]^ Previous studies have reported a 12 to 80% incidence of moderate-to-severe postoperative pain.^[8,9]^ Postoperative pain represents a significant postoperative stressor for patients. In the absence of adequate treatment in a timely manner, it has the potential to impede the rapid postoperative recovery process, prolong hospitalization, and increase the burden on healthcare resources.^[10,11]^ Furthermore, the severity of acute postoperative pain is strongly correlated with the likelihood of developing a persistent chronic pain state.^[12]^ This has implications for the patient’s recovery process and may also result in a long-term reduction in quality of life. Despite the availability of local anesthetics, opioids and cyclooxygenase inhibitors for pain control during and after surgery, clinical evidence indicates that postoperative pain in obese patients remains a significant clinical challenge. Consequently, there is an urgent need for the development of individualized assessment scales for the precise prevention and treatment of postoperative pain.

Kalkman and colleagues developed a composite scoring system based on age, gender, type of surgery, preoperative pain level, and anxiety level to predict the severity of pain in the early postoperative period.^[13]^ However, it should be noted that the present scoring system is not applicable to all types of surgery, nor does it address the specific population of obese patients. Prior to being applicable in different types of surgery, it still needs to be validated through the use of large-scale cohort studies. In light of the aforementioned background, the objective of this study was to delve deeper into the efficacious predictive factors influencing postoperative pain and to elucidate the risk factors associated with moderate-to-severe postoperative pain in patients undergoing LSG. This would enable the identification of high-risk patients preoperatively, thereby facilitating the formulation of bespoke pain prevention and management plans. This, in turn, would enhance the clinical outcome and the patients’ postoperative quality of life.

## 2. Materials and methods

### 2.1. Materials

#### 2.1.1. Materials collection

We used a medical big data system to include 375 consecutive patients who were admitted to Jinan Central Hospital and underwent LSG for obesity with complete medical records from January 2017 to June 2023, and recorded the patients’ postoperative Numeric Rating Scale (NRS) scores within 24 hours, with 0 being no pain and 10 being the most severe pain. Included patients with the highest NRS score greater than or equal to 4 within 24 hours were defined as the moderate-severe pain group, and patients with the highest NRS score <4 within 24 hours were defined as the non-moderate-severe pain group. General information [(including age, sex, systolic blood pressure (SBP), diastolic blood pressure (DBP), height, weight, body mass index (BMI), etc)] and clinical information (coagulation routine, blood routine, liver and kidney function, blood glucose, blood electrolytes, blood lipids, etc) were collected from both groups. Six circumferences (chest circumference, neck circumference, abdominal circumference, thigh circumference, upper arm circumference, hip circumference), and intraoperative blood gas analysis within half an hour were recorded in detail for each patient, and PONV was collected by the postoperative follow-up team of the anesthesia department. Weight loss surgery at our center is performed by a regular anesthetic and surgical team, and the anesthetic regimen is maintained consistently in all cases.

#### 2.1.2. Anesthesia protocol and drug’s name

Each patient was admitted to the operating room with the peripheral vein open, connected to cardiac monitoring, nasal catheter oxygenation, and radial artery puncture catheterization under local anesthesia for continuous monitoring of invasive arterial pressure. An 8 mg dose of the antiemetic drug ondansetron was administered prior to induction, and a fast-track intravenous induction intubation technique was employed. This involved the administration of propofol at a dose of 2 mg/kg, and sufentanil at a dose of 0.3 to 0.5 µg/kg in sequence. Following the disappearance of the patient’s eyelash reflex, a 1 mg/kg dose of rocuronium bromide was administered. The aforementioned induction drug dosages were calculated based on the lean body weight (LBW).^[14]^ Following 90 to 120 seconds of mechanical ventilation via face mask, the patient was intubated with a transoral plain view tracheal tube under visual laryngoscopic guidance. Mechanical ventilation was initiated, and the partial pressure of end-expiratory carbon dioxide (PETCO₂) was monitored. Additionally, the patient’s bispectral index was continuously monitored by connecting to the electroencephalogram dual-frequency index meter. Combined intravenous and inhalation anesthesia for intraoperative anesthesia maintenance: sevoflurane (2–3%) and propofol (3–4 mg per kilogram per hour) with continuous pumping of remifentanil (0.1–0.2 μg per kilogram per minute). The maintenance doses of these agents were calculated according to the adjusted body weight (ABW).^[14]^ During the surgical procedure, a specific depth of anesthesia was maintained (bispectral index value of 40–50). When insufficient analgesia was identified (the patient’s heart rate increased by more than 15% of the basal heart rate, and other potential causes were excluded). A dosage of 10 µg of sufentanil was administered, and intravenous rocuronium bromide was injected to maintain inotropic relaxation. Additional drugs were subsequently added by the anesthesiologist in accordance with the evolving clinical situation. Furthermore, the anesthesiologist adjusted the intravenous injection or pumping of vasoactive drugs in response to changes in the patient’s blood pressure, heart rate, and other vital signs. The administration of sevoflurane was terminated prior to the closure of the incision, while intravenous anesthesia was discontinued at the conclusion of the surgical procedure. Subsequently, Sugammadex (Bridion) 2 mg/kg (ABW) was administered to antagonize the myorelaxation effects. An intravenous analgesia pump was connected postoperatively (sufentanil 1.5 μg/kg, saline diluted to 100 mL, set background infusion rate 2 mL/h). The endotracheal tube was removed when the patient was awake and met the criteria for extubation, and the patient was transferred to the post-anesthesia recovery unit for resuscitation. Once the patient’s Steward score (out of 6) had reached 5 or above and met the criteria for discharge from the post-anesthesia recovery unit, the patient was transferred back to the ward to continue their treatment. The routine use of remedial analgesic drugs was not indicated in cases where the patient’s need for analgesia (NRS ≤ 3) was met within 24 hours following surgery. However, in instances where the patient’s pain level (NRS > 3) remained unmanaged, the case was registered and the patient was referred for remedial analgesia. The gastrointestinal surgeon then implemented the necessary remedial analgesic measures, taking into account the specific circumstances of the patient. Total body weight (TBW): the actual weight of the patient. LBW: LBW (kg, women) = 9270 × TBW/(8780 + (244 × BMI (kg.m^-2^)), LBW (kg, men) = 9270 × TBW/(6680 + (216 × BMI (kg.m^−2^)), ideal body weight (IBW): IBW (kg) = height (cm) − x (where x = 105 in females and 100 in males), adjusted body weight (ABW): ABW (kg) = (IBW (kg) + 0.4 (TBW (kg) − IBW (kg)). The study protocol was approved by the Ethics Committee of Jinan Central Hospital, and the data were exported using processing methods such as the removal of sensitive personal information. The study protocol secured approval from the Ethics Committee of Jinan Central Hospital (ethical number: 2023-075-01), and precautions were taken to anonymize sensitive personal information during data export, there was no invasion of privacy and interference with patient care in this study.

#### 2.1.3. Inclusion criteria

(1) American Society of Anesthesiologists classification I to III, gender, and age; (2) patients who underwent LSG for obesity, type 2 diabetes or metabolic syndrome; (3) cases with complete clinical case data.

#### 2.1.4. Exclusion criteria

(1) Patients with comorbid psychiatric disorders and other diseases that cause communication difficulties and inability to cooperate; (2) patients with incomplete clinical medical records, with the number of missing modified frailty index (mFI) items ≥ 1, and missing BMI records; (3) patients whose medical records affect their privacy; (4) patients who experience intraoperative malignant events; (5) patients with a history of alcohol or drug addiction; (6) patients experiencing pain due to other acute or chronic diseases; patients with a preoperative NRS score of zero above; (7) patients who have been taking analgesics for an extended period prior to surgery; (8) patients whose anesthesia records do not align with the specified anesthesia protocols.

#### 2.1.5. Grouping criteria

The postoperative follow-up results provided by the postoperative follow-up team of the Department of Anesthesiology in our hospital and the NRS scores in the medical record system for differentiation were used to categories patients into 2 groups: those with moderate-to-severe pain within the first 24 hours after surgery and those with less severe pain. (1) The moderate-severe pain group (P group) comprised patients who had an NRS score of 4 or above within 24 hours of surgery. (2) The non-moderate-severe pain group (NP group) consisted of patients who had an NRS score of <4 within the subsequent 24 hours.

### 2.2. Methods

#### 2.2.1. General information collection

The medical history section of the medical big data system was utilized to extract pertinent information from patients undergoing LSG operations. This included age, height, weight, history of smoking, history of alcohol consumption, and past medical history.

#### 2.2.2. Frailty assessment

The mFI was selected for assessment based on the medical big data system. In this study, the mFI was analyzed using the total score method, whereby each item of the mFI represents 1 point, and the mFI score is equal to the total number of deficiencies present. mFI values reflect the degree of frailty of the patient, with larger values indicating a greater degree of fragmentation and more health deficiencies present in the patient. The mFI comprises a total of 11 entries, as follows: (1) history of diabetes mellitus; (2) preoperative independent functional status (nonindependent); (3) chronic obstructive pulmonary or pneumonitis; (4) history of congestive heart failure; (5) history of myocardial infarction within 6 months; (6) history of percutaneous coronary intervention/cardiac stent implantation/angina pectoris; (7) history of hypertension requiring medication; (8) history of peripheral vascular disease/ischemic rest pain; (9) history of impaired sensory function; (10) history of transient ischemic attack or cerebrovascular accident; (11) history of cerebrovascular accident with neurological impairment.

#### 2.2.3. Surgical method

LSG operation points: the operation is performed with the patient in the lying position. The main surgeon and the mirror holder are located on the right side of the patient, while the assistant is located on the left side. The pneumoperitoneum needle is used to establish the pneumoperitoneum, and the five-hole method is employed. The roving nurse then inserts the 36Fr gastric correction tube (Bougie) through the mouth and empties the stomach. The gastric colonic ligaments are then dissected using an ultrasonic knife in the gastric omental vascular arches along the gastric wall, with the fundus, greater curvature, and posterior gastric wall being completely freed. The roving nurse assisted in fixing a gastric correction tube to the antrum as a guide for sleeve gastric dissection. The start of dissection was initiated at a distance of approximately 4 cm from the pylorus, with the first shot performed at a distance of approximately 3 cm from the gastric angle. The last shot was recommended to be performed at a distance of 2.0 cm from the angle of His. The staple line was closed with continuous sutures of 3-0 absorbable sutures, and the greater omentum was secured to the side of the greater curvature of the residual stomach.

#### 2.2.4. Statistical methods

The data were analyzed using SPSS 25.0 and R (4.2.1). For the measurements, those that conformed to a normal distribution were expressed as mean ± standard deviation and analyzed using the *t* test. Those that did not conform to a normal distribution were expressed using the interquartile method and analyzed using the *U* test. For the counts, they were expressed as percentages and analyzed using the chi-square test or Fisher exact probability method. The results were analyzed using the following statistical tests: univariate and multivariate logistic regression analyses were employed to ascertain the risk factors for moderate-to-severe postoperative pain, with the diagnostic efficacy of the pertinent indices evaluated by plotting the receiver operating characteristic (ROC) curves. A simplified scoring system was devised on the basis of the findings of the multifactorial logistic regression analysis, and risk stratification was conducted on the basis of the sum of the scores attributed to various variables. A *P* value of <.01 was determined to be statistically significant.

## 3. Results

### 3.1. Comparison of general data

A total of 375 patients were included in this study, comprising 189 patients in the P group and 186 patients in the NP group. A *T* test was employed to assess the general data of the 2 populations among the 375 patients who underwent LSG (Table 1). The results demonstrated that there was no significant difference between the 2 groups with respect to gender (male), SBP, and operation time. The mean values for SBP, diastolic blood pressure (DBP), and operation time were found to be comparable between the 2 groups. The 2 groups differed significantly in age, smoking status, and alcohol consumption, previous bariatric surgery, obstructive sleep apnea syndrome, BMI, chest circumference, waist circumference, hip circumference, upper arm circumference, thigh circumference, neck circumference, the observed difference was statistically significant (*P* < .05).

**Table 1:** Comparison of general data of patients in P group and NP group.

	NP group (n = 186)	P group (n = 189)	*P* value
Sex (male, n%)	85 (45.7%)	104 (55.0%)	.071*
Age (years)	42.75 ± 12.62	53.51 ± 15.08	<.001**
SBP (mm Hg)	130.81 ± 10.68	132.44 ± 13.42	.190
DBP (mm Hg)	80.24 ± 7.40	80.64 ± 11.90	.690
Smoking (n%)	25 (13.4%)	41 (21.7%)	.036
Drinking (n%)	27 (14.5%)	41 (21.7%)	.010*
History of bariatric surgery (n%)	15 (8.1%)	3 (1.6%)	.003*
PONV (n%)	130 (69.9%)	85 (45.0%)	<.001**
OSAS (n%)	27 (14.5%)	13 (6.9%)	.017*
BMI (kg/m^2^)	32.45 ± 3.27	41.79 ± 6.98	<.001**
Chest circumference (mm)	110.70 ± 7.80	127.34 ± 11.86	<.001**
Waist circumference (mm)	105.21 ± 13.40	128.50 ± 16.08	<.001**
Hip circumference (mm)	115.41 ± 9.17	132.20 ± 14.80	<.001**
Upper arm circumference (mm)	34.17 ± 4.29	38.76 ± 5.66	<.001**
Thigh circumference (mm)	60.56 ± 7.22	67.3 ± 8.81	<.001**
Neck circumference (mm)	39.14 ± 3.20	43.33 ± 4.40	<.001**
Surgery time (min)	111.78 ± 25.60	116.63 ± 25.89	.069
Intraoperative remifentanil consumption [μg/(kg min)]	0.11 ± 0.01	0.11 ± 0.02	.638
Intraoperative sufentanil consumption (μg)	81.46 ± 10.83	86.55 ± 10.86	.437
mFI (n%)			<.001**
0	37 (19.9%)	14 (7.4%)	
1	74 (39.8%)	56 (29.6%)	
2	71 (38.2%)	58 ((30.7%)	
3	3 (1.6%)	47 (24.9%)	
4	1 (0.5%)	12 (6.3%)	
5	0 (0.0%)	1 (0.5%)	
6	0 (0.0%)	1 (0.5%)	
ASA (n%)			.128
1	15 (8.1%)	13 (6.9%)	
2	170 (91.4%)	168 (88.9%)	
3	1 (0.5%)	8 ((4.2%)	

The data are presented as mean ± SD for continuous variables and n for categorical variables.

ASA = American Society of Anesthesiologists, BMI = body mass index, DBP = diastolic blood pressure, mFi = modified frailty index, NP = non-moderate-to-severe pain, OSAS = obstructive sleep apnea syndrome, P = moderate-to-severe pain, PONV = postoperative nausea and vomiting, SBP = systolic blood pressure.

* *P* < .05.

** *P* < .01.

### 3.2. Comparison of biochemical indicators

The general biochemical parameters included in this study were white blood cell, red blood cell, neutrophil, platelet, alanine aminotransferase, aspartate aminotransferase, direct bilirubin, albumin, triglyceride, total cholesterol, free fatty acid, low-density lipoprotein cholesterol, and high-density lipoprotein cholesterol, lactate dehydrogenase, alkaline phosphatase, uric acid, cystatin, creatine kinase, potassium, sodium. *T* test was used to compare the general data of the 2 populations in 375 patients who underwent LSG, and the results showed that in Table 2.

**Table 2:** Comparison of laboratory indexes of P group and NP group.

	NP (n = 186)	P (n = 189)	*P* value
WBC (×10^9^/L)	7.72 ± 1.87	8.21 ± 2.06	.016*
RBC (×10^12^/L)	4.51 ± 0.45	4.8 ± 0.58	<.001**
Neut (×10^9^/L)	4.43 ± 1.52	4.81 ± 1.68	.020*
PLT (×10^9^/L)	288.49 ± 67.53	280.28 ± 66.23	.236
ALT (IU/L)	30.42 ± 27.78	43.35 ± 37.23	<.001**
AST (IU/L)	21.03 ± 12.23	25.99 ± 16.2	.001*
DBIL (g/L)	3.37 ± 1.72	3.81 ± 2.34	.036*
ALB (g/L)	44.19 ± 4.38	42.98 ± 4.32	.007*
TG (mmol/L)	1.79 ± 1.49	1.95 ± 1.58	.319
TC (mmol/L)	4.48 ± 1.22	4.59 ± 1.1	.335
Apo (mmol/L)	25.71 ± 26.91	27.54 ± 26.68	.509
LDL (mmol/L)	2.68 ± 0.92	2.73 ± 0.8	.569
HDL (mmol/L)	1.1 ± 0.36	1.03 ± 0.28	.051
LDH (U/L)	188.54 ± 58.43	202.9 ± 57.33	.017*
ALP (g/L)	68.75 ± 20.89	76.06 ± 21.54	.001*
UA (μmol/L)	343.04 ± 111.98	415.43 ± 139.18	<.001**
CysC (mg/L)	0.7 ± 0.29	0.77 ± 0.32	.039*
Scr (μmol/L)	66.69 ± 91.67	63.17 ± 24.33	.610
CK (U/L)	84.03 ± 51.64	100.98 ± 60.31	.004*
K + (mmol/L)	3.79 ± 0.99	3.87 ± 0.89	.441
Na + (mmol/L)	138.39 ± 15.09	138.34 ± 18.39	.978
Ph	7.37 ± 0.05	7.36 ± 0.06	.211
PaCO_2_ (Pa)	39.77 ± 5.37	41.96 ± 5.93	<.001**
PaO_2_ (Pa)	284.32 ± 97.45	226.36 ± 90.52	<.001**
Carbon dioxide concentration (mmol/L)	24.03 ± 1.85	24.91 ± 2.2	<.001**

The data are presented as mean ± SD for continuous variables and n for categorical variables.

ALB = albumin, ALP = alkaline phosphatase, ALT = alanine aminotransferase, AST = aspartate aminotransferase, CK = creatine kinase, CysC = cystatin, DBIL = direct bilirubin, FFA = free fatty acid, HDL = high-density lipoprotein cholesterol, K+ = potassium, LDH = lactate dehydrogenase, LDL = low-density lipoprotein cholesterol, Na^+^ = sodium, Neut = neutrophil, NP = non-moderate to severe pain, P = moderate to severe pain, PLT = platelet, RBC = red blood cell, TC = total cholesterol, TG = triglyceride, UA = uric acid, WBC = white blood cell.

* *P* < .05.

** *P* < .01.

### 3.3. Single factor logistic regression analysis

Single factor logistic regression analysis of the indicators with significant differences derived from the *T* test and *U* test showed that smoking history, obstructive sleep apnea syndrome, BMI, chest circumference, waist circumference, hip circumference, and upper arm circumference, thigh circumference, neck circumference, mFI, and red blood cell, alanine aminotransferase, aspartate aminotransferase, uric acid, creatine kinase, and whole blood carbon dioxide concentration were the risk factors for moderate-to-severe postoperative pain. But a history of bariatric surgery was a protective factor for moderate-to-severe postoperative pain (Table 3).

**Table 3:** Single factor logistic regression analysis.

	B	*P* value	Exp (B)	95% CI
Age (years)	0.053	<.001**	1.054	1.038 ± 1.070
History of bariatric surgery (n%)	-1.694	.008**	0.184	0.052 ± 0.646
PONV (n%)	1.044	<.001**	2.840	1.858 ± 4.343
BMI (kg/m^2^)	0.402	<.001**	1.495	1.379 ± 1.621
chest circumference (mm)	0.179	<.001**	1.195	1.154 ± 1.239
waist circumference (mm)	0.122	<.001**	1.13	1.102 ± 1.158
hip circumference (mm)	0.119	<.001**	1.127	1.098 ± 1.156
upper arm circumference (mm)	0.233	<.001**	1.263	1.190 ± 1.340
thigh circumference (mm)	0.110	<.001**	1.117	1.082 ± 1.152
neck circumference (mm)	0.318	<.001**	1.374	1.272 ± 1.484
mFI (n%)	0.799	<.001**	2.223	1.746 ± 2.830
RBC (×10^12^/L)	1.115	<.001**	3.050	1.967 ± 4.730
ALT (IU/L)	0.013	<.001**	1.013	1.006 ± 1.020
AST (IU/L)	0.025	.001*	1.026	1.01 ± 1.041
ALP (g/L)	0.016	.001*	1.017	1.007 ± 1.027
UA (μmol/L)	0.005	<.001**	1.005	1.003 ± 1.007
CK (U/L)	0.006	.005*	1.006	1.002 ± 1.009
PaCO_2_ (Pa)	0.069	<.001**	1.072	1.032 ± 1.112
PaO_2_ (Pa)	-0.006	<.001**	0.994	0.991 ± 0.996
Carbon dioxide concentration (mmol/L)	0.216	<.001**	1.242	1.118 ± 1.379

ALT = alanine aminotransferase, ALP = alkaline phosphatase, AST = aspartate aminotransferase, BMI = body mass index, CK = creatine kinase, NP = non-moderate to severe pain, P = moderate to severe pain, PONV = postoperative nausea and vomiting, mF = modified frailty index, RBC = red blood cell, UA = uric acid.

* *P* < .05.

** *P* < .01.

### 3.4. Multivariate logistic regression analysis

The predictors of moderate-to-severe pain after LSG were analyzed with the occurrence of moderate-to-severe pain after LSG as the dependent variable, and the risk factors and protective factors identified in the univariate analysis as the independent variables age, BMI, and mFI were found to be the independent risk factors for moderate-to-severe pain after LSG details were shown in Table 4

**Table 4:** Multivariate logistic regression analysis.

	B	*P* value	Exp (B)	95% CI
Age (years)	0.078	<.001**	1.081	1.045 ± 1.119
History of bariatric surgery (n%)	0.917	.364	2.502	0.345 ± 18.116
PONV (n%)	0.805	.084	2.237	0.897 ± 5.582
BMI (kg/m^2^)	0.528	<.001**	1.696	1.363 ± 2.111
chest circumference (mm)	0.058	.138	1.059	0.982 ± 1.143
waist circumference (mm)	0.025	.318	1.025	0.977 ± 1.075
hip circumference (mm)	-0.028	.438	0.972	0.905 ± 1.044
upper arm circumference (mm)	-0.087	.139	0.917	0.818 ± 1.028
thigh circumference (mm)	0.028	.457	1.029	0.955 ± 1.108
neck circumference (mm)	0.059	.418	1.061	0.919 ± 1.224
mFI (n%)	1.384	<.001**	3.993	2.284 ± 6.979
RBC (×10^12^/L)	-0.129	.797	0.879	0.330 ± 2.340
ALT (IU/L)	0.011	.515	1.012	0.977 ± 1.047
AST (IU/L)	-0.004	.927	0.996	0.922 ± 1.077
ALP (g/L)	-0.004	.697	0.996	0.973 ± 1.018
UA (μmol/L)	0.002	.424	1.002	0.997 ± 1.006
CK (U/L)	-0.003	.435	0.997	0.989 ± 1.005
PaCO_2_ (Pa)	-0.105	.014*	0.901	0.828 ± 0.979
PaO_2_ (Pa)	-0.002	.549	0.998	0.994 ± 1.003
Carbon dioxide concentration (mmol/L)	-0.033	.785	0.967	0.763 ± 1.227

ALT = alanine aminotransferase, ALP = alkaline phosphatase, AST = aspartate aminotransferase, BMI = body mass index, CK = creatine kinase, mFI = modified frailty index, NP = non-moderate to severe pain, P = moderate to severe pain, PONV = postoperative nausea and vomiting, RBC = red blood cell, UA = uric acid.

* *P* < .05.

** *P* < .01.

### 3.5. Nomograms and verification

**Figure 1.**
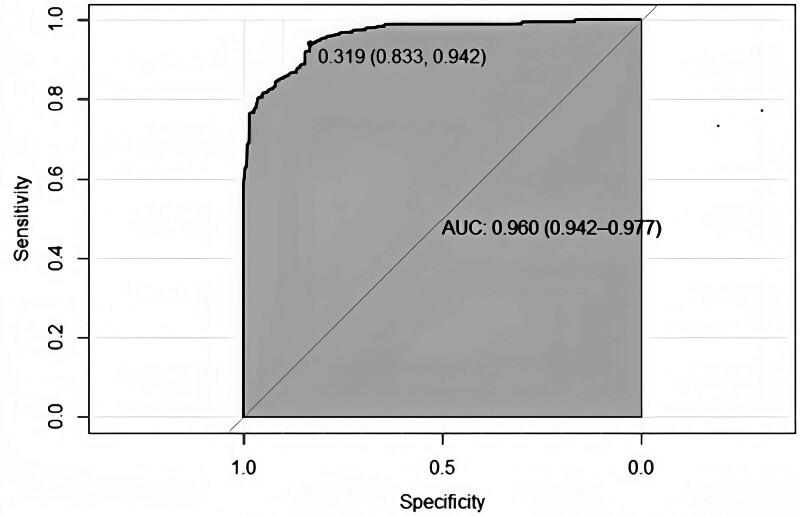
The ROC curve plot. The area under curve of the predictive model was 0.96 (95% CI: 0.942–0.977) with sensitivity and specificity of 94.2% and 83.3% in the ROC curve plot. ROC = receiver operating characteristic.

The model equation for predicting moderate-to-severe pain after LSG, which incorporates 3 independent risk factors, is as follows: Y = (-27.322) + 0.074*(Age) + 0.496*(BMI) + 1.391*(mFI). The model prediction probability was plotted on a ROC curve, and the area under the curve was 0.96 (95% CI: 0.942–0.977). This yielded a sensitivity of 94.2%, a specificity of 83.3%, and a maximum value of the Yoden index of 0.775 (Fig. 1).

**Figure 2.**
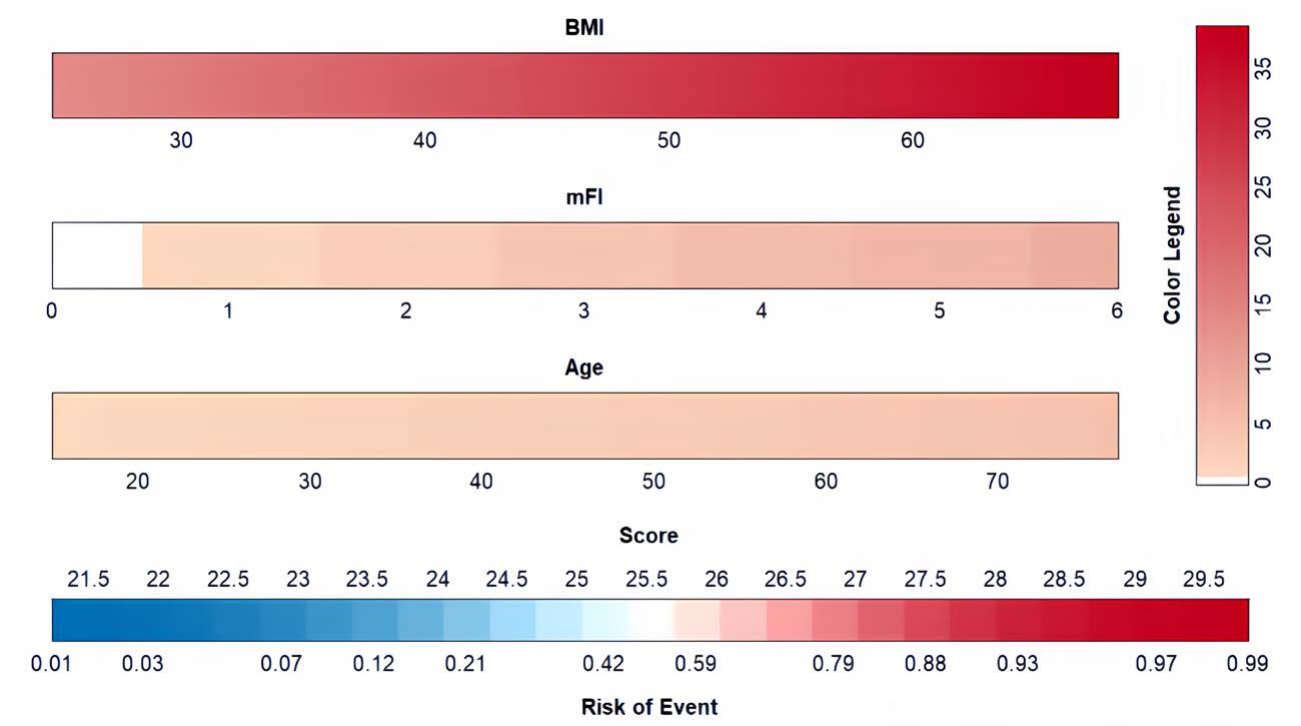
Nomogram plot. BMI = body mass index, mFI = modify frailty index.

The resulting model was further simplified for scoring purposes. The visualization model was constructed by assigning a certain score to each independent variable according to the magnitude of its regression coefficient (Fig. 2). In this column-line graph, BMI was the largest predictor of moderate-to-severe pain after LSG (100 points), followed by mFI (30 points), and age (15 points).

**Figure 3.**
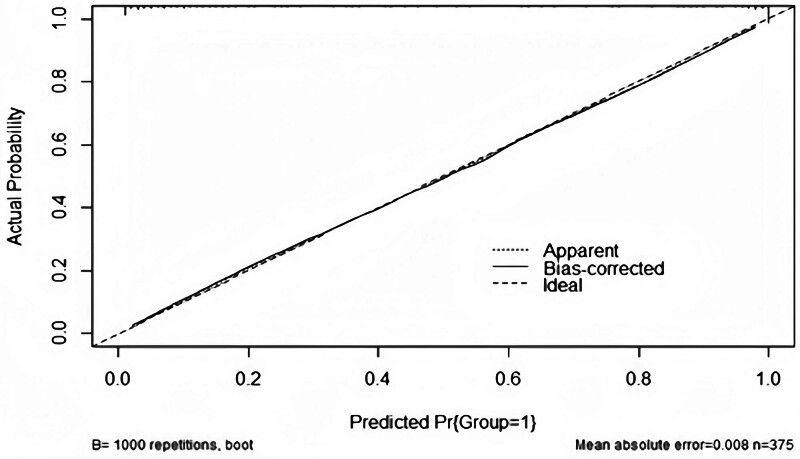
Calibration plot of the training set. The predictive models had good calibration with a mean error of 0.008.

An internal validation method was employed to plot a calibration curve using the bootstrap method, which demonstrated that the mean difference between predicted and actual values was 0.008 (Fig. 3).

**Figure 4.**
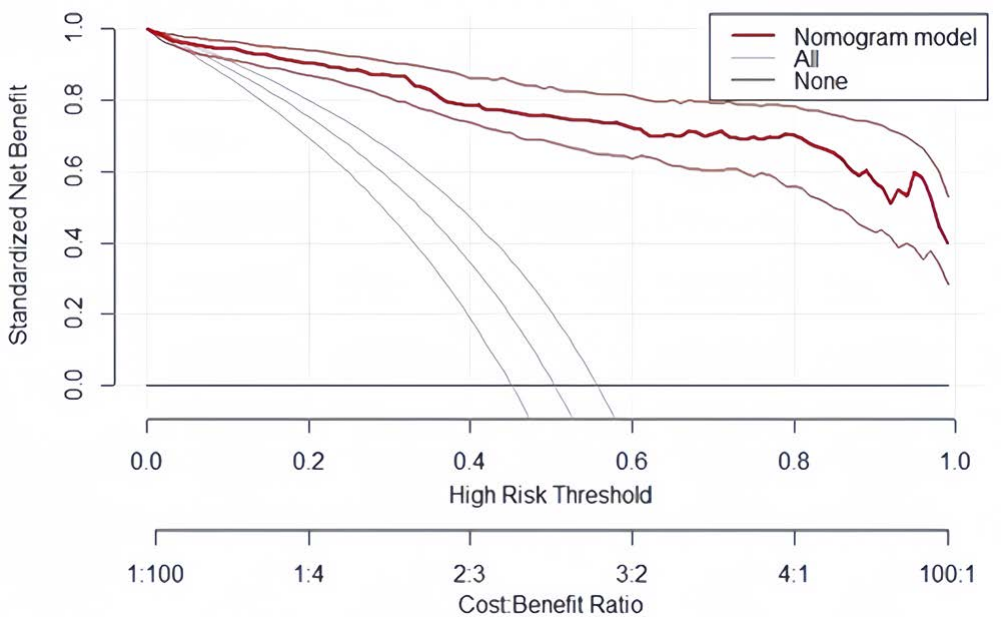
Clinical decision analysis curve. The clinical decision analysis curve showed that using the model to predict the development of PONV produced more benefit when the probability of PONV between 0.001 and 0.999. PONV = postoperative nausea and vomiting.

The clinical decision analysis curves demonstrate that the model exhibits a positive benefit when the threshold probability is between 0.001 and 0.999 (Fig. 4).

### 3.6. Model accuracy

To better assess the accuracy of the model, we further categorize patients based on their NRS scores as follows: mild pain (NRS 1–3), moderate pain (NRS 4–6), and severe pain (NRS 7–10). Subsequently, we apply the model to patients with moderate and severe NRS scores respectively to determine the accuracy of the model.

#### 3.6.1. Moderate pain

**Figure 5.**
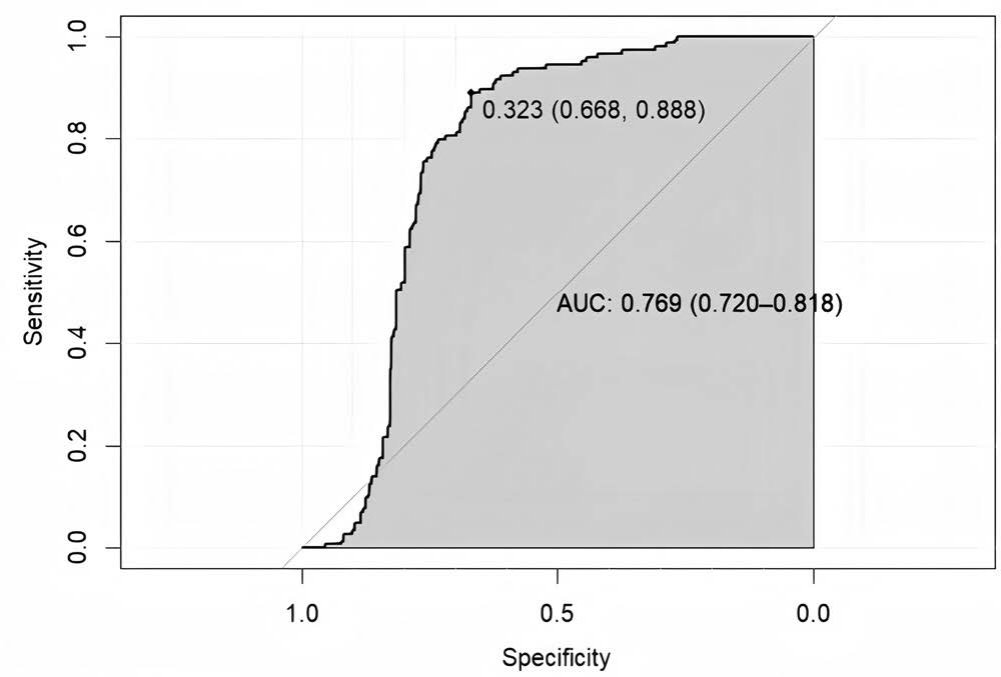
The ROC curve plot. The area under curve of the predictive model was 0.769 (95% CI: 0.720–0.818) with the sensitivity and specificity of 88.8% and 66.8% in the ROC curve plot, ROC = receiver operating characteristic.

The model equation for predicting moderate pain after LSG, which incorporates 3 independent risk factors: BMI, mFI, age. The model prediction probability was plotted on a ROC curve, and the area under the curve was 0.769 (95% CI: 0.720–0.818). This yielded a sensitivity of 88.8%, a specificity of 66.8%, and a maximum value of the Yoden index of 0.556 (Fig. 5).

**Figure 6.**
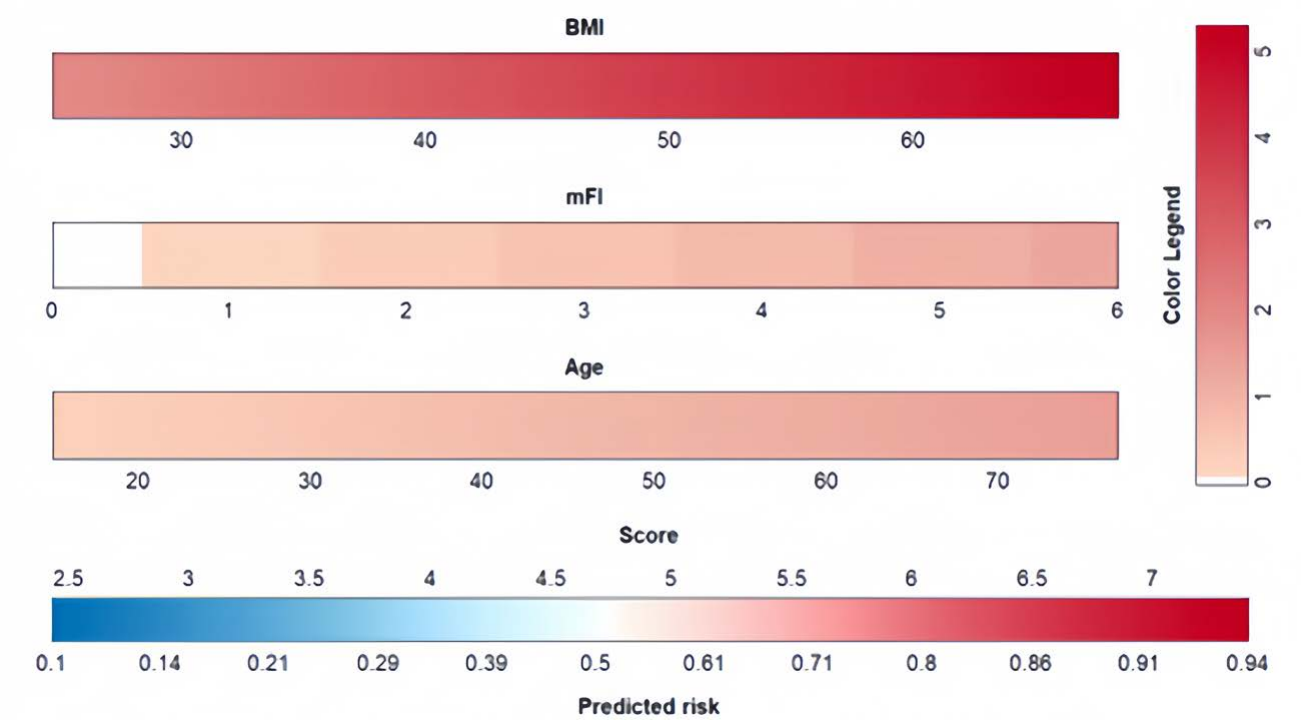
Nomogram plot. BMI = body mass index, mFI = modify frailty index.

The resulting model was further simplified for scoring purposes. The visualization model was constructed by assigning a certain score to each independent variable according to the magnitude of its regression coefficient (Fig. 6). In this column-line graph, BMI was the largest predictor of moderate-to-severe pain after LSG (100 points), followed by mFI (34 points), and age (33 points).

#### 3.6.2. Severe pain

**Figure 7.**
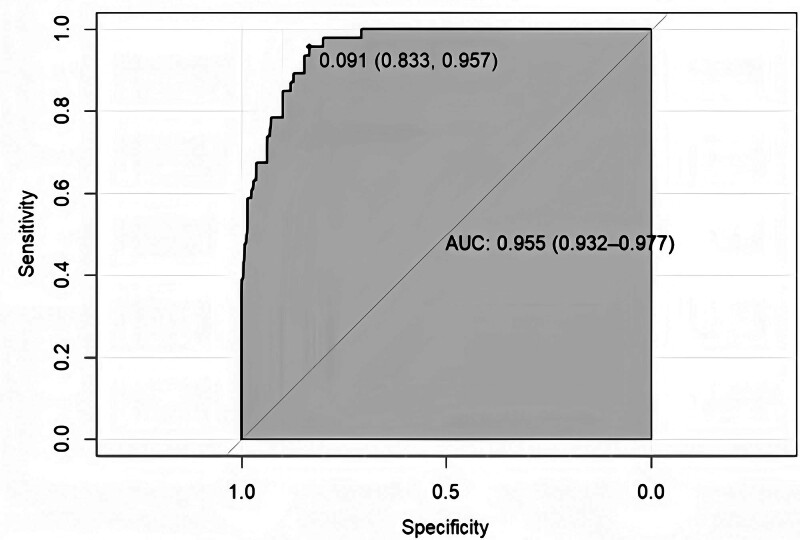
The ROC curve plot. The area under curve of the predictive model was 0.955 (95% CI: 0.932–0.977) with the sensitivity and specificity of 95.7% and 83.3% in the ROC curve plot. ROC = receiver operating characteristic.

The model equation for predicting severe pain after LSG, which incorporates 3 independent risk factors: BMI, mFI, age. The model prediction probability was plotted on a ROC curve, and the area under the curve was 0.955 (95% CI: 0.932–0.977). This yielded a sensitivity of 95.7%, a specificity of 83.3%, and a maximum value of the Yoden index of 0.790 (Fig. 7).

**Figure 8.**
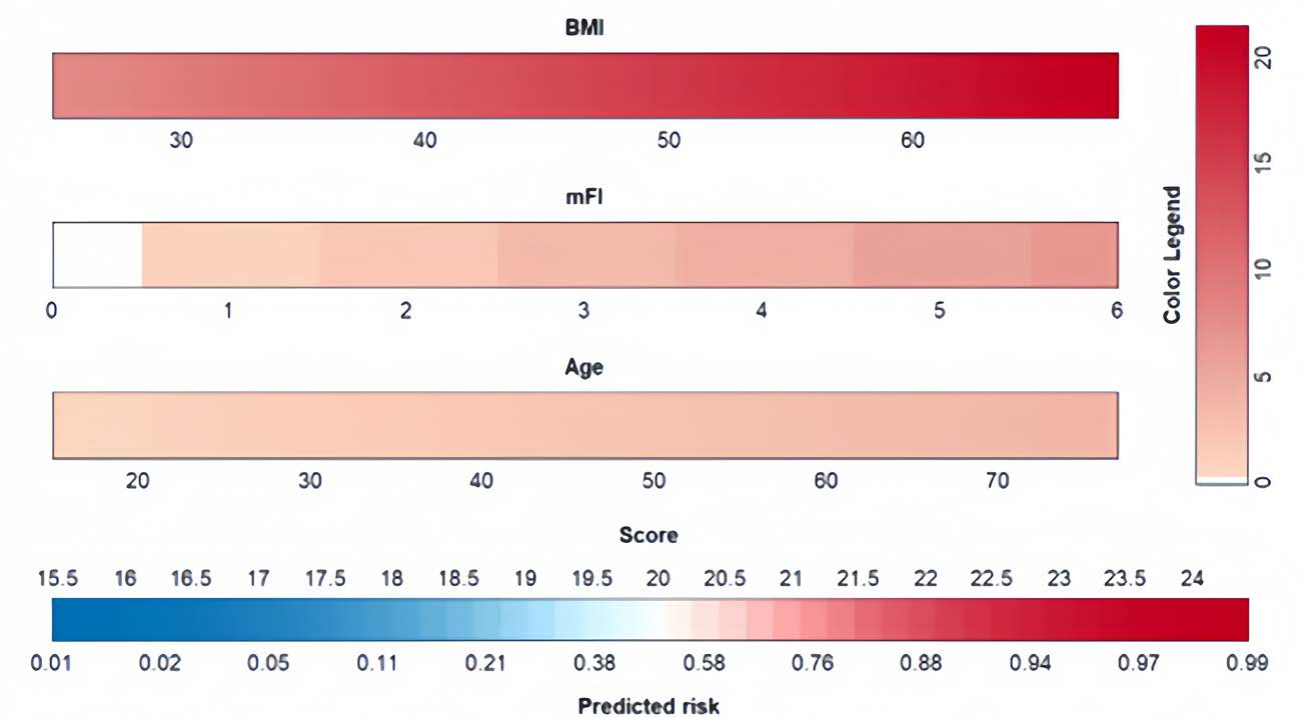
Nomogram plot. BMI = body mass index, mFI = modify frailty index.

The resulting model was further simplified for scoring purposes. The visualization model was constructed by assigning a certain score to each independent variable according to the magnitude of its regression coefficient (Fig. 8). In this column-line graph, BMI was the largest predictor of moderate-to-severe pain after LSG (100 points), followed by mFI (43 points), and age (21 points).

## 4. Discussion

The findings of this study indicate that age, BMI, and a mFI are the primary predictors of moderate-to-severe pain following LSG. Based on these key indicators, a predictive model was constructed with the objective of assessing the risk of moderate-to-severe pain in obese patients undergoing LSG. Validation of the model using calibration curves demonstrated high predictive accuracy, with a mean absolute error of only 0.008. This provides clinicians with a powerful tool to more accurately predict and manage postoperative pain.

As the global prevalence of obesity continues to rise, there has been a notable increase in the number of weight loss surgeries, particularly LSG surgeries.^[3,4]^ The utilization of a rational analgesic regimen during surgical procedures is of paramount importance in order to minimize the occurrence of postoperative pain and complications.^[15]^ The under- or overtreatment of postoperative pain can result in a range of adverse outcomes, including increased postoperative mortality, increased hospitalization costs, increased complications, and possibly even chronic pain.^[12,16]^ In patients who are obese and undergoing LSG surgery, postoperative pain not only leads to the aforementioned problems but can also cause serious adverse events such as PONV.^[17]^

A substantial body of research has demonstrated a robust correlation between BMI)an d pain sensitivity.^[18]^ A higher BMI is not only associated with an increased self-perception of pain, but is also strongly associated with pain-related disability, mental health problems and reduced physical functioning.^[19–21]^ The intricate nature of this relationship is exemplified by the multitude of interactions, encompassing a convergence of pain-processing mechanisms within the central nervous system and inflammatory pathways throughout the body.^[22–24]^ A substantial body of evidence has emerged indicating a notable rise in the prevalence of nonsteroidal antiinflammatory drug utilization among individuals with obesity. This phenomenon may be associated with their elevated baseline pain levels and heightened necessity for pain management.^[25,26]^ While nonsteroidal antiinflammatory drugs are effective in relieving mild to moderate pain, their long-term use may result in gastrointestinal and cardiovascular side effects, which adds complexity to pain management in obese patients. The retrospective analysis of this study further corroborated the significant correlation between BMI and the occurrence of moderate-to-severe postoperative pain, thereby underscoring the pivotal role of BMI as a risk factor for postoperative pain. Despite the consideration of additional body circumference indicators, including waist circumference and chest circumference, these were found to be less significant than BMI in predicting postoperative pain.

Furthermore, our study found that mFI is a significant risk factor for the onset of moderate-to-severe postoperative pain in patients undergoing LSG surgery. Frailty is a medical syndrome characterized by a reduction in strength, endurance, and physiological reserve.^[27]^ In the past, frailty has been regarded as a wasting disorder, typically observed in older adults who are small in stature and thin in build.^[28,29]^ Nevertheless, contemporary research indicates that frailty may co-exist with obesity, a condition designated as “obesity-frailty syndrome.”^[30,31]^ This finding is of particular importance for obese patients, who may experience debility even in the absence of advanced age. The mFI, a widely utilized assessment instrument in clinical practice, has been demonstrated to predict the incidence of postoperative complications associated with bariatric surgery.^[32]^ Furthermore, preoperative debilitating states have been linked to the development of chronic postoperative pain. Patients experiencing these states are more susceptible to heightened postoperative pain, diminished daily functionality, and reduced physical activity.^[33]^ It has been demonstrated that frailty is associated with the development of chronic pain following major elective noncardiac surgery in a surgical population.^[34]^ Consequently, the identification of preoperative frailty characteristics can inform the implementation of interventions designed to enhance surgical outcomes and improve patients’ postoperative quality of life. In obese patients undergoing bariatric surgery, it is crucial to identify preoperative frailty and to rationalize the use of perioperative analgesics in order to reduce postoperative pain.

Moreover, age emerged as a significant predictor of postoperative pain in this study. It has been demonstrated that individuals with obesity may encounter heightened psychosocial challenges, including anxiety and fear, which may precipitate or exacerbate postoperative pain.^[35]^ Furthermore, obese individuals are at an elevated risk of developing cardiovascular and endocrine disorders with age.^[36]^ These chronic conditions have a significant impact on the overall health of the patient, and may also result in an increase in debilitating scores, which can exacerbate postoperative pain. It is therefore essential that these factors are given due consideration in the context of both the preoperative assessment and the postoperative management plan, in order to facilitate the development of an individualized pain management plan.

In conclusion, the prediction model employed in this study incorporates key variables such as age, BMI, and mFI, which are supported by robust theoretical evidence and demonstrate robust predictive efficacy, this study sought to identify the risk factors for moderate-to-severe postoperative pain following LSG. Using these identified factors, a prediction model was developed and validated in patients with moderate and severe postoperative pain. Model validation yielded favorable results, providing clinical staff with high-risk factors for perioperative analgesia in patients undergoing LSG, and patients with more comfortable, safe, and personalized treatment. Nevertheless, this study is not without limitations. Firstly, as a single-center retrospective study, there is a possibility of incomplete clinical data, which may impact the accuracy of the study results. The impact of this limitation was mitigated by the exclusion of patients with extensive missing data and the utilization of a multiple interpolation method to address the remaining incomplete observations. Secondly, the study population was limited to adult patients who elected to undergo LSG, and thus the results may not be generalizable to the wider bariatric surgery population. Thirdly, although we have adjusted for potential confounding variables, there may still be unconsidered variables, such as surgical incision size and postoperative care, which may also have an impact on the study results.

To conclude, the predictive model created in this study, based on BMI, mFI and age, offers a valuable point of reference for anticipating moderate-to-severe pain following LSG in obese patients. Further validation and optimization of this model are required to enhance its value and generalizability in clinical practice.

## Author contributions

**Data curation:** Chengzhen Zhang, Xiaoqian Yu.

**Funding acquisition:** Wenying Chi.

**Methodology:** Kaiyun Zhang.

**Project administration:** Fanjun Meng.

**Resources:** Wenying Chi.

**Software:** Bin Zheng.

**Validation:** Guo Junzuo.

**Writing – review & editing:** Yaning Yang, Xiaoqian Yu.
